# Gammaproteobacteria as essential primary symbionts in the striped shield bug, *Graphosoma Lineatum* (Hemiptera: Pentatomidae)

**DOI:** 10.1038/srep33168

**Published:** 2016-09-09

**Authors:** Naeime Karamipour, Mohammad Mehrabadi, Yaghoub Fathipour

**Affiliations:** 1 Department of Entomology, Faculty of Agriculture, Tarbiat Modares University, Tehran, Iran

## Abstract

Many members of suborder Heteroptra harbor heritable symbiotic bacteria. Here we characterize the gut symbiotic bacterium in *Graphosoma lineatum* (Hemiptera: Pentatomidae) by using molecular phylogeny, real-time PCR analysis as well as light and electron microscopy observations. The microscopy observations revealed the presence of a large number of rod-shaped bacterial cells in the crypts. A very high prevalence (98 to 100%) of the symbiont infection was found in the insect populations that strongly supports an intimate association between these two organisms. Real-time PCR analysis also showed that the Gammaproteobacteria dominated the crypts. The sequences of *16sr RNA* and *groEL* genes of symbiont showed high levels of similarity (93 to 95%) to *Pantoea agglomeranse* and *Erwinia herbicola* Gammaproteobacteria. Phylogenetic analyses placed *G. lineatum* symbiont in a well-defined branch, divergent from other stink bug bacterial symbionts. Co-evolutionary analysis showed lack of host-symbiont phylogenetic congruence. Surface sterilization of eggs resulted in increased pre-adult stage in the offspring (aposymbionts) in comparison to the normal. Also, fecundity, longevity, and adult stage were significantly decreased in the aposymbionts. Therefore, it seems that the symbiont might play a vital function in the host biology, in which host optimal development depends on the symbiont.

Many insects possess symbiotic bacteria inside their body, particularly those feeding on restricted diets, such as plant sap, vertebrate blood, or woody material^1^. These symbiotic associations span a spectrum of types that differ with respect to the effect of the symbiont on the host^2^. Symbionts assist their insect hosts in various functions such as providing essential nutrients^3^, defending from natural enemy^4^,^5^, increasing host resistance against unfavorable environmental conditions^6^, and detoxifying insecticides^7^. Of the various symbiotic associations, the most intimate forms are found in obligate associations, in which insect hosts rely on intracellular bacterial symbionts for their development and fecundity^8^. Typical characteristics of these bacterial symbionts are vertical transmission to the next generations, co-speciation between the insect host and its bacterial symbionts^8^,^9^, increasing of genome AT content, genome size reduction and fast sequence evolution^3^,^10^,^11^. On the other hand, in facultative symbiotic associations, insects are independent of bacterial symbiont for their survival^12^.

Among different orders of insects, Hemiptera, have particularly developed special association with bacterial symbionts. Most of these insects feed on poor-quality food with low nutritional value^10^,^13^. Phytophagous stinkbugs, for example, harbor bacterial symbionts in the fourth section of their midgut. This section of gut has special structures (crypts) that are colonized by extracellular bacterial symbionts^14^. In stinkbugs, extracellular gut symbionts are vertically transmitted by post-hatching transmission mechanisms such as egg surface contamination, coprophagy^15^ or by formation and deposition of maternal special symbiont-containing capsules^16^. In spite of being extracellular, genome reduction and missing some of the genes that are necessary for free living bacteria have been evolved in these symbionts^17^,^18^,^19^,^20^. Besides, co-speciation between the insect host and the symbiont in some of the stink bugs family^17^,^20^,^21^, indicated that these symbiotic relationships were important for the insect host and bacterial symbionts. Moreover, experimental removing of symbionts from insects have caused retarded growth, mortality, sterility as well as morphological abnormalities^16^,^21^,^22^,^23^,^24^,^25^. On the other hand, the symbiotic beneficial functions have caused the host to provide different conditions for maintaining and transmission of symbionts such as modified midgut crypts and vertical transmission to the next generation. These host physiological and behavioral adaptations for maintenance of symbionts provide further evidence of co-evolution and strict relationships between hosts and their symbionts^14^.

In the present study, we explored the primary gut symbiont of *G. lineatum* and its importance in the insect biology. We report the presence of a crypt-dwelling gram-negative bacterium (Gammaproteobacteria) in the insect. This symbiotic bacterium is AT-rich, transmitted vertically through egg surface contamination. Egg surface sterilization to prevent symbiotic bacterial transmission resulted in less fecundity and longevity of the insect host in comparison to normal insects indicating essential role of the symbionts in the insect host.

## Results

### General observations of *G. lineatum* related to transmission and acquisition of its bacterial symbiont

**Figure 1:**
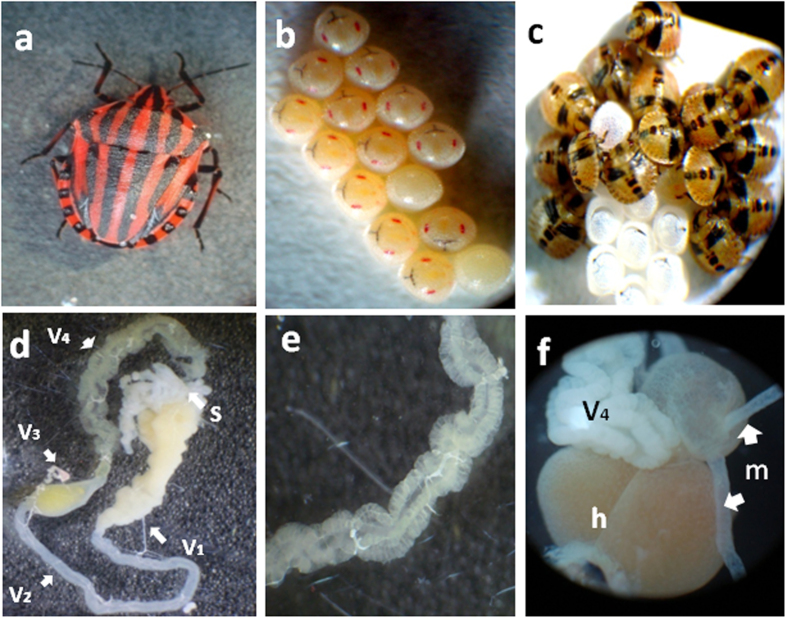
Morphological features of *G. lineatum*. (**a**) adult insect, (**b**) egg mass (**c**) newly hatched nymphs that stay together and aggregate on the eggshell (**d**) midgut from an adult female of *G. lineatum*; V1, first section of midgut ; V2, second section of midgut; V3, third section of midgut; V4, fourth section of midgut; S, salivary gland, (**e**) midgut fourth section (V4) with crypts; (**f**) connection of midgut fourth section to the hindgut, h: hindgut, m: malpighian tubules.

Adult females of *G. lineatum* (Fig. 1a) were observed to deposit about 14 eggs per clutch. The eggs hatched within six or seven days (Fig. 1b). The newly hatched nymphs were observed to aggregate on the eggshell for about two days, probably because of probing of the egg surface for acquisition of the symbiont that already occurred during maternal transmission (Fig. 1c). The insect midgut was composed of four different sections. The first section of midgut was a sac-like organ filled with liquid material; the second section was long and tubular; the third section of midgut was soft and slightly expanded (Fig. 1d); and the fourth section was long and white in color with four rows of well-developed crypts (Fig. 1e). The fourth section was extended to the region where the malpighian tubules originated (Fig. 1f).

### Light microscopy and TEM of the midgut crypts

**Figure 2:**
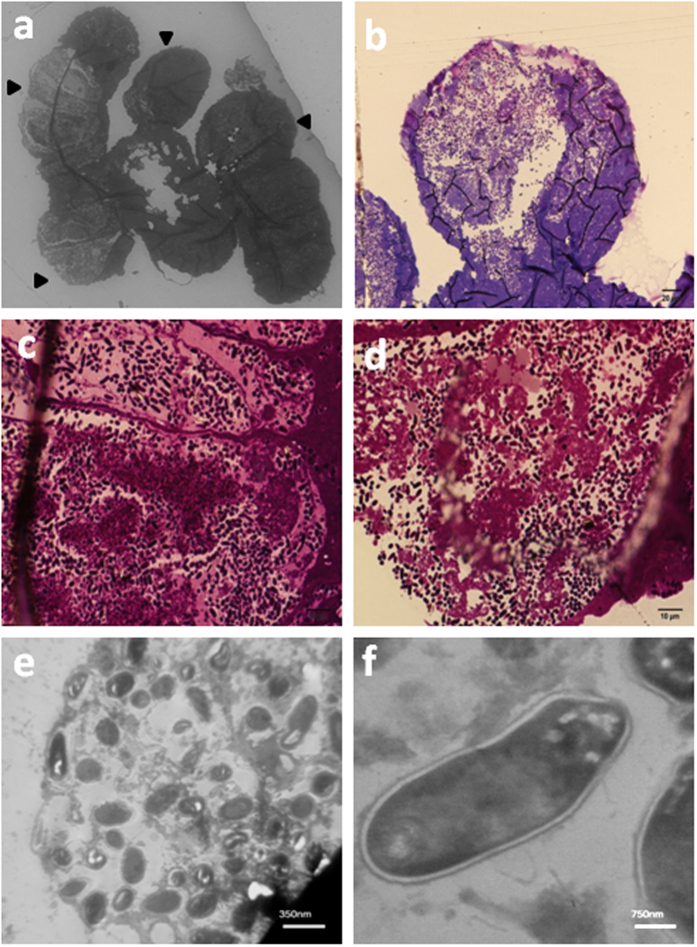
Light microscopy and transmission electron microscopy (TEM) of *G. lineatum* crypts. (**a**–**d**) Light microscopy images from enclosed crypt; a large number of bacteria as observed in the crypts. L: lumen. (**e**) TEM of a crypt containing symbiotic bacteria. (**f**) TEM of a single symbiotic bacterium in the crypt.

In order to see bacterial symbiont cells, we prepared thin transverse cross sections from the fourth midgut section of *G. lineatum*. Light microscopy (Fig. 2a–d) and TEM (Fig. 2e,f) observations revealed that a large number of rod-shape bacterial cells (≈1 μm) were harbored in the crypts of midgut. The bacterial symbionts were morphologically uniform bearing well-developed cell walls with two membranes, typical of gram-negative bacteria, but without obvious flagellum (Fig. 2c). Crypts were enclosed from the gut lumen, thereby maintaining the symbiont in the isolated cryptic cavities.

### Prevalence and frequency of the bacterial symbiont in the insect host

**Figure 3:**
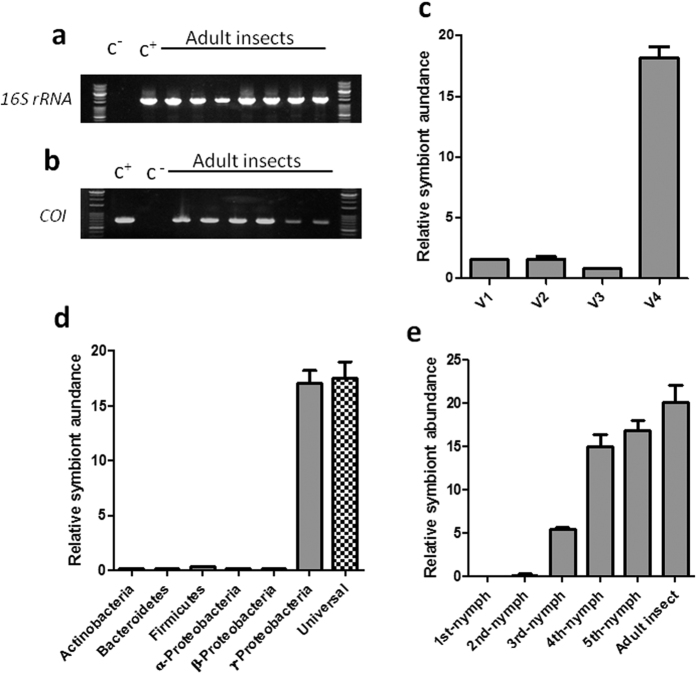
Amplification of conserved genes of the symbiont and *G. lineatum*. (**a**) Detection of symbiotic bacteria with *16S rRNA* gene in adult individuals. (**b**) *COI* gene of the *G. lineatum*. (**c**) Relative quantification of the bacterial symbionts in different developmental stages of *G. lineatum*. (**d**) Composition of bacteria within the V4. (**e**) Relative quantification of the bacterial symbionts in different developmental stages of *G.* *lineatum*. c-: negative control [distilled water], c+: positive control [DNA from midgut crypts of *G. lineatum* that harbored symbionts].

To evaluate the prevalence of the bacterial symbiont in *G. lineatum*, a total of 100 adult insects and 10 individuals of each nymphal stage were subjected to PCR symbiont detection based on *16S rRNA* gene. *COI* gene of insects was also amplified by using specific primers. Our results showed that nearly all (98–100%) the adult insects harbored the bacterial symbiont (Fig. 3a). The *COI* genes of adult insects were also amplified and sequenced (Fig. 3b).

The relative abundance of bacteria was compared along the insect midgut by using qPCR and eubacterial primers. The results showed that the forth midgut section (V4) which contained crypts, harbored high density of bacteria in comparison to other regions (Fig. 3c). To assess the composition of bacteria within the V4, different taxon-specific primers were used for qPCR assays. The Gammaproteobacteria dominated the communities in the V4, whereas the Alphaproteobacteria, the Betaproteobacteria, the Actinobacteria and the Firmicutes were not detected or detected at the very low levels (Fig. 3d).

To estimate relative density of the bacterial symbiont in different developmental stages of the insect, we used specific qPCR primers. Stage-specific detection of bacterial symbionts showed that the first and second nymphal stages had the lowest population of the symbiont; however, the bacterial population highly increased thereafter and reached the highest population in the adult insects (Fig. 3e).

### Phylogenetic placement of the symbiotic bacteria based on 16S rRNA and groEL gene

**Figure 4:**
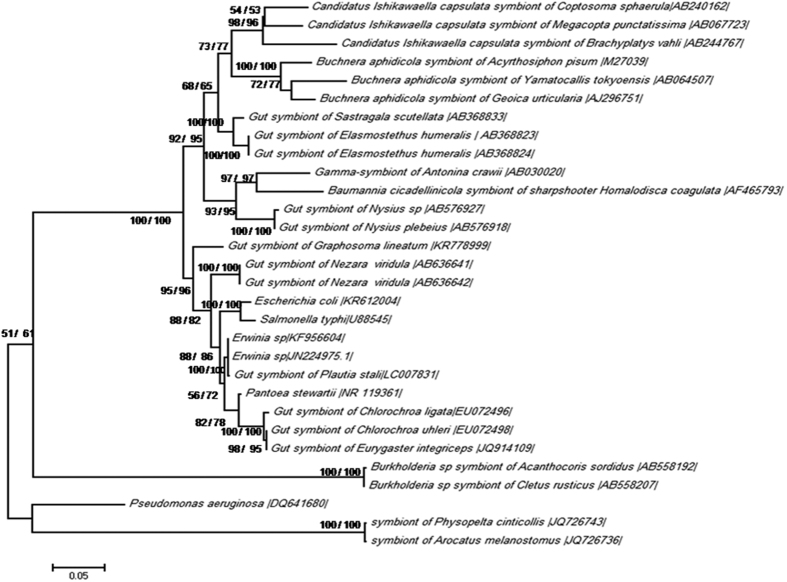
Phylogenetic placement of the symbiotic bacterium based on the *16S rRNA* gene. ML: maximum likelihood method with 100 replications based on GTR + G + I model. NJ: neighbor-joining method with 1000 replications. On the nodes, bootstrap probabilities neighbor-joining/maximum likelihood analysis are shown. Accession numbers of sequences are shown in brackets.

**Figure 5:**
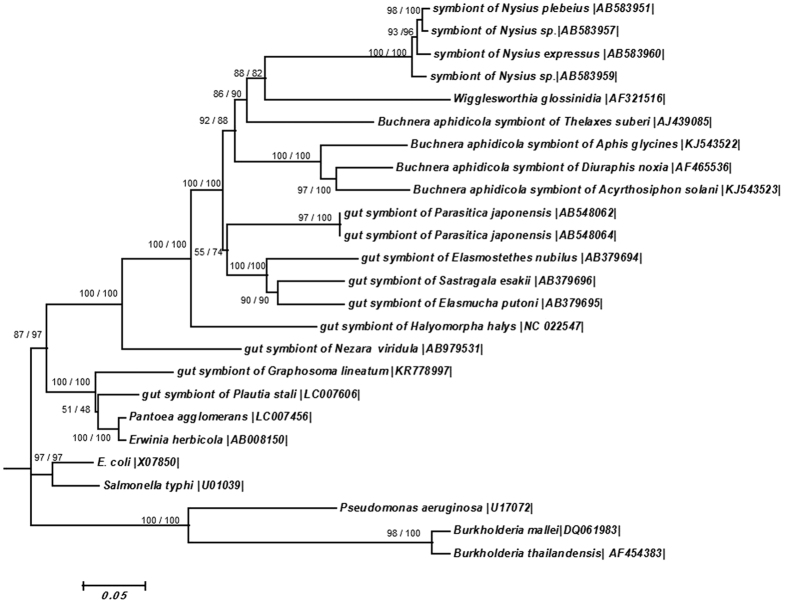
Phylogenetic placement of symbiotic bacteria based on the *groEL* gene. ML: maximum likelihood method with 100 replication based on GTR model. NJ: neighbor-joining method with 1000 replications. On the nodes, bootstrap probabilities neighbor-joining/maximum likelihood analysis are shown. Accession numbers of sequences are shown in brackets.

**Figure 6:**
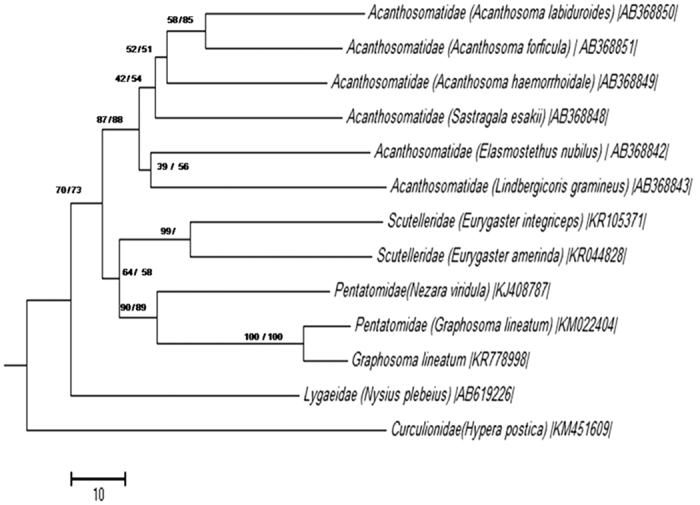
Phylogenetic placement of *G. lineatum* based on *COI* gene. ML: maximum likelihood method with 100 replications based on GTR + G + I model. NJ: neighbor-joining method with 1000 replications. On the nodes are the bootstrap probabilities neighbor-joining/maximum likelihood analysis are shown. Accession numbers of sequences are shown in brackets.

BLAST (Basic Local Alignment Search Tool) analyses of *16S rRNA* and *groEL* gene sequences exhibited the most similarity with *Erwinia herbicola* [AB008150] (with 95% and 92% identity for *16S rRNA* and *groEL* gene, respectively) and *Pantoea agglomeranse* [LC007456] (with 95% and 93% identity for *16S rRNA* and *groEL* gene, respectively). AT contents were 47.45% for *16S rRNA* on 1452 bp and *groEL* gene AT contents were 47.03% on 1516 bp. The gene sequences were subjected to molecular phylogenetic analyses together with *16S rRNA* and *groEL* gene sequences of other insect symbionts. In phylogenetic trees based on *16S rRNA* genes, the *G. lineatum* symbiont was placed with the bacterial symbionts of Pentatomidae and Scutelleridae bugs in a clade with high bootstrapping values (Fig. 4). In *groEL* phylogenetic trees of different bacterial symbionts, the *G. lineatum* symbiont grouped with bacterial symbionts of Pentatomidae, as well (Fig. 5). The phylogenies of the insect host deduced based on *COI* sequence data placed *G. lineatum* within Pentatomidae clade with high bootstrap support value (Fig. 6).

### Co-evolution between *G. lineatum* and its bacterial symbiont

**Figure 7:**
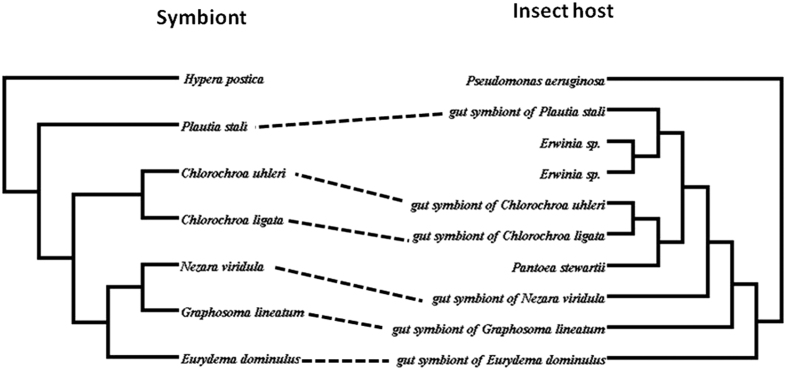
Co-evolutionary analysis of *G. lineatum* and the symbiont. Tanglegram of *16S rRNA* tree of symbiont and *COI* tree of *G. lineatum* that showed incongruence between the insect host tree and the symbiont tree as the Icong index was insignificant (p = 0.85).

To determine if the phylogeny of the symbiont mirrored the insect host phylogeny, we compared the phylogeny of *G. lineatum* inferred from *COI* gene sequence with the phylogeny of its endosymbiont inferred from bacterial *16S rRNA*. The phylogenies of the bacterial symbiont and the insect host deduced from the sequence data showed lack of congruency between the trees (Fig. 7). The Icong congruence index for testing topological similarity between trees also revealed that the trees were not more congruent than expected by chance (P-value of 0.85).

### The symbiont transmission via egg surface contamination

**Figure 8:**
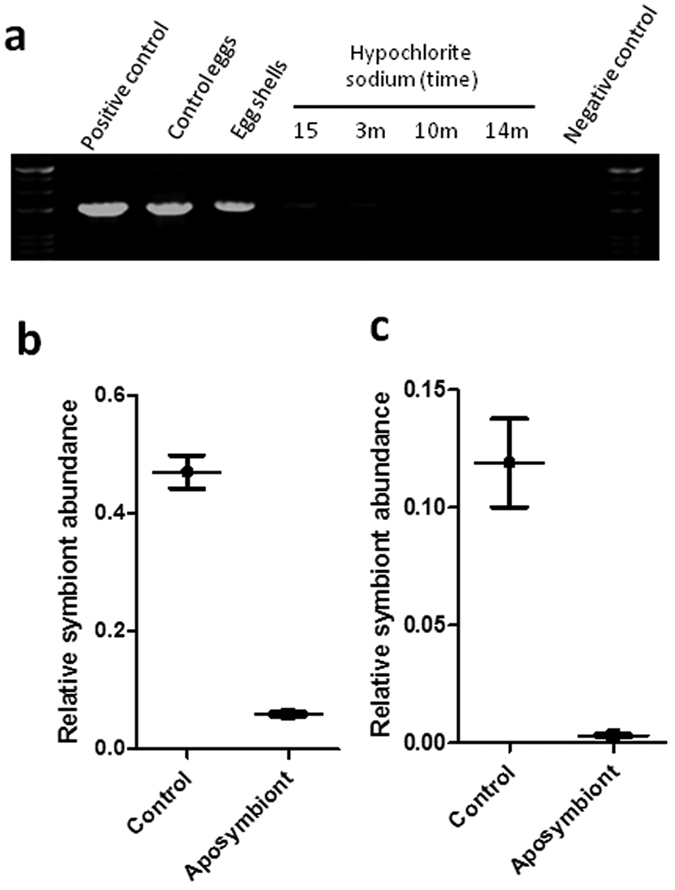
Egg surface sterilization for elimination of the symbiotic bacterium. (**a**) Surface sterilization of *G. lineatum* eggs with hypochlorite sodium to eliminate bacterial symbionts. Bacterial population declined after sterilization of eggs (**b**) qPCR analysis of DNA extracted from untreated eggs (control) and surface-sterilized eggs of *G. lineatum*. Bacterial symbiont population dramatically declined after sterilization of eggs (**c**) qPCR analysis for bacterial symbiont intensity in first nymphs of *G. lineatum* hatched from control and surface-sterilized eggs with hypochlorite sodium for 15 s. Egg sterilization decreased bacterial symbiont intensity in treated nymphs in comparison with the controls.

Given that we demonstrated the constant presence of the symbiont in the crypts of *G. lineatum*, it was envisaged that there must be an efficient transmission route for delivering bacterial symbiont to the offspring. Since the most common symbiont transmission mode in true bugs is through egg surface contamination, different surface sterilization methods were used to see if the symbiont would be eliminated. DNA was extracted from each egg mass separately, and subjected to PCR detection. The results showed that ethanol treatment couldn’t eliminate the symbiont. Washing the eggs with 12% hypochlorite sodium for 14 min almost completely eliminated the bacterial symbiont, however, the egg hatching rate was quite low, reaching nearly zero (Data not shown). To improve the egg hatching rate, the washing time was cut short which resulted in less stringent sterilization (Fig. 8a), but enhanced the hatching rate almost to the level of normal eggs. These results confirmed that vertical transmission of symbiont to the progeny is only via egg surface contamination. To further confirm reduction of the bacterial symbionts in the aposymbiont insects, we estimated the relative density of the bacterial symbionts in the aposymbiont and the control insect. Our results indicate that density of the bacterial symbiont in the sterilized eggs was dramatically reduced when compared to the control eggs (Fig. 8b). The level of symbiont density in aposymbiont first-instars was about 38 folds lower than that of control insects (Fig. 8c).

### Removal of bacterial symbiont negatively affects host biology

**Figure 9:**
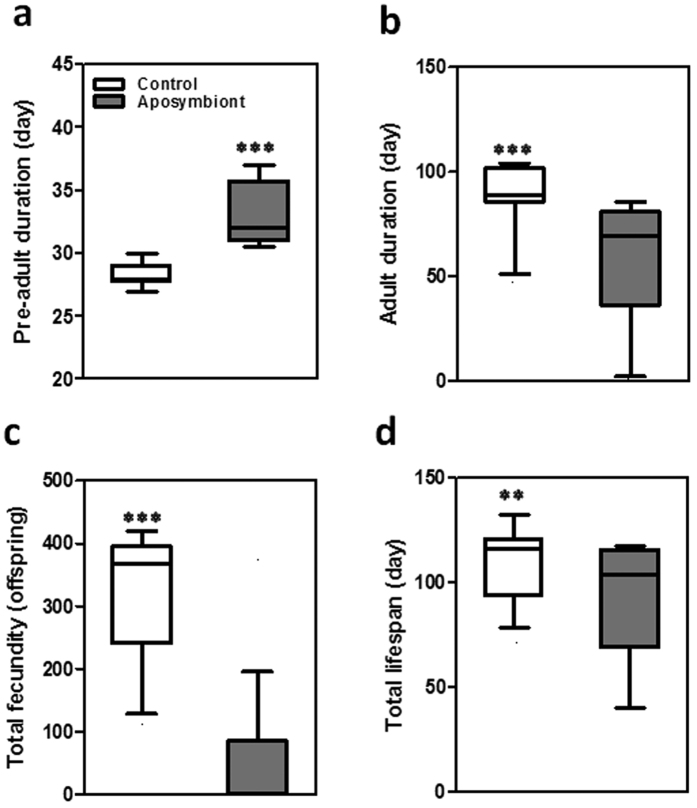
Impact of bacterial symbiont elimination on the host fitness. (**a**) The pre-adult duration, (**b**) the adult stage duration, (**c**) total life span, and (**d**) fecundity in aposymbiont and control insects. Shading of boxes denotes the experimental treatment (the aposymbiont insects). Lines represent medians, boxes comprise the 10–90 percentile, and whiskers denote the range. Asterisks indicate a significant difference between the control and the aposymbiont insects (Kruskal-Wallis test, P < 0.0001).

Considering the high prevalence of the symbiont and its efficient vertical transmission, we questioned whether the symbiont has an important role in the insect host. To test this hypothesis, we sterilized the eggs by 12% hypochlorite sodium for 15 seconds. We already had checked efficiency of this procedure for elimination of the symbiont and we found that this method didn’t affect hatching rate while other methods highly decreased hatching rate (data not shown); therefore, this treatment was used for egg sterilization (Fig. 8b). The sterilized egg masses were kept until emergence of second instar nymphs; then, every individual was isolated in a separate container until they developed to adults. The adult insects were paired and allowed to reproduce. Different biological parameters were measured for both the control and aposymbiont insects. Our results indicated significant changes in most of the developmental parameters between the control and aposymbiont insects. Durations of pre-adult stages were significantly increased in the aposymbiont insects (32.87 ± 0.54 day) in comparison to that of control insects (28.50 ± 1.50 days) (Fig. 9a). Also, in the control insect, fecundity and total life span as well as duration of adult stage were 2.0, 1.2 and 1.4 folds higher than those of aposymbiont insects, respectively (Fig. 9b–d). These findings highlight the important role of the symbiont in *G. lineatum*.

## Discussion

Many true bugs harbor bacterial symbionts in their midgut crypts. The most important symbionts in these insects are extracellular bacteria that live in crypts around the terminal region of the midgut. These symbionts belong to the Gammaproteobacteria and are present in different families of true bugs such as Pentatomidae^23^,^26^,^27^,^28^, Plataspidae^16^, Cydnidae^29^, Acanthosomatidae^21^ and Scutelleridae^30^,^31^. In this study, we investigated the gut symbiont of *G. lineatum*, a member of Pentatomidae stinkbugs. This insect harbored gram-negative bacteria in their symbiotic organs (crypts that placed around the midgut fourth section). We found that the crypts harbored highly numbers of a specific Gammaproteobacterium that was consistently present during the insect development. These results showed that the symbiont might have an important function in the insect host. BLAST analyses of *16S rRNA* and *groEL* gene sequences from this bacterium showed high similarity with plant-dwelling bacteria in the genera *Erwinia* and *Pantoea*. Phylogenetic analyses of these bacterial genes indicated that the symbiont was related to bacterial symbionts of Pentatomomorpha bugs. It has been suggested that the gut symbionts of pentatomid bugs are polyphyletic with multiple evolutionary origins^32^,^33^ as we found in the present study. Dynamic evolutionary trajectories of pentatomid gut symbionts have recently been experimentally demonstrated^34^.

Insect symbionts are usually well adapted to living inside the body. Consequently, genome of these symbionts are AT-rich, reduced in size, and show fast evolution^3^,^10^,^11^. In *G. lineatum* light and transmission electron microscopy showed a large number of intercellular rod-shaped symbiotic bacteria having organized cell wall that were placed in enclosed crypts. Moreover, the bacterial symbiont *16S rRNA* and *groEL* genes showed slight AT bias.

Given that insect gut symbionts are extracellular bacteria, post hatching vertical transmission is a normal phenomenon in these symbionts. We showed that the symbiont bacteria of *G. lineatum* are vertically transmitted post hatching via egg surface contamination. New born nymphs stay on the egg shell for about two days, which might be for acquisition of the symbiont. This behavior has been reported from other stink bugs^15^,^21^,^27^,^35^. Therefore, it seems that egg-surface probing in true bugs is a conserved behavior in nymphs that warrants vertical transmission of the symbionts. This mode of symbiont transmission, however, is not perfect as the egg surface is prone to contamination by the environmental bacteria and symbiont replacement. Therefore, it seems that egg smearing strategy is an intermediate mode of symbiont transmission between strict capsule-transmitted gut symbiotic bacterium in plataspid stinkbugs^16^ and environmental acquisition of *Burkholderia* in alydids without vertical transmission^36^.

The history of associations between insect hosts and their symbionts were studied in different investigations. In extracellular gut symbionts of stinkbugs, strict host-symbiotic associations have been reported in Acanthosomatidae^21^, Plataspidae^20^ and Urostylididae^17^ bugs. Our co-phylogeny analysis showed lack of significant congruency between the true bugs and the bacterial symbionts. Phylogeny of *G. lineatum* based on *COI* gene wasn’t mirrored with its bacterial symbiont phylogeny *16S rRNA*. These results provided further evidences for Polyphyly of gut symbionts in this insect.

Host-symbiont co-evolution and vertical transmission of symbionts indicate that the symbionts have important roles in insect hosts^2^,^8^. Therefore, experimental removal of obligate symbiont might negatively affect the insect host. In some cases, elimination of gut symbionts of true bugs, causes mortality and delay in developmental time within their host^16^,^21^,^22^. To test if the gut symbiotic bacterium contributes in *G. lineatum* homeostasis, we sterilized *G. lineatum* egg surface. In some of stink bugs, this method cannot fully remove symbiont and may just reduce the number of bacterial symbiont^37^. In our study, long exposure of egg masses to hypochlorite sodium highly decreased the rate of egg hatching to almost near zero. It is noted that just after three min washing in hypochlorite sodium, the egg masses were separated and the rate of hatching highly declined. We showed that a 15 second washing in hypochlorite sodium resulted in normal hatching rate and produced aposymbiotic insects. The aposymbiotic insects showed lower growth and fecundity in comparison to the normal insects that highlighted the contribution of this symbiotic bacterium in *G. lineatum* biology. Most of the symbionts have been shown to have nutritional roles in their host. Like most other true bugs, the active feeding stage of *G. lineatum* is the second-instar nymph; interestingly, we observed that high mortality and delay in growth occurred in the second-instar that is congruent with recent findings on *Sibaria englemani*^33^. Therefore, it seems that the symbiotic bacterium of *G. lineatum* might have vital role in provision of essential nutrients necessary to support host survival, development and fecundity. However, more studies are needed to prove this hypothesis.

In conclusion, we have shown that *G. lineatum* harbored a primary symbiotic bacterium in the crypts that belongs to gram-negative gammaproteobacterium. This crypt-dwelling symbiotic bacterium was consistently detected in geographically distinct *G. lineatum* populations that strongly support a close association between these two organisms. Our findings also suggest that the symbiotic bacterium transmitted vertically by egg surface contamination. Finally, we showed that egg surface sterilization interfered with the insect host biology. On the whole, our results demonstrate that *G. lineatum* and its symbiont might have evolved together and the insect host is dependent on the bacterial symbiont for normal growth and development.

### Experimental procedures

#### Insects

In the wild, *G. lineatum* are found in the north of Iran and feed on umbelliferous plants including *Foeniculum vulgare*. Populations of *G. lineatum* were received from Varamin Agriculture Research Center (Varamin, Iran), which, were previously collected from different areas of Central Alborz, Iran, during summer 2014. The insects were transferred to the laboratory and placed within rearing cages (30 × 30 × 20 cm) to establish a laboratory colony. *G. lineatum* individuals were held in a growth chamber (temperature of 27 ± 1 °C, 60 ± 5% relative humidity [RH] and photoperiod of 16:8 h [Light: Dark]) and were provided the seeds of *F. vulgare* and water.

#### DNA extraction and PCR

In order to extract DNA from the insect hosts and the bacterial symbionts, the insects were surface sterilized and the digestive organs were dissected. The forth section of the midgut (V4) containing crypts was isolated for further analysis. DNA was extracted from the isolated tissues by homogenization them in a lysis buffer (10 mM Tris-HCl, 10 mM EDTA, and 10% SDS, pH8.0). Proteinase K (final concentration of 0.25 μg/μl) was added and the samples were incubated at 40 °C for two hours and then extracted with phenol/chloroform. DNA was precipitated by adding 2 volumes of ethanol and 0.2 volume of 3 M sodium acetate, pH 5.3, and centrifugation at 15,000 × g for 20 min. The pellet was washed with 70% ethanol, dried at 37 °C, and re-suspended in dsH2O^38^. The quality of extracted DNA was assessed by running 5 μl of DNA on 1% agarose gel. Concentrations of DNA samples were also quantified and adjusted to the same concentration.

The DNA samples were subjected to PCR amplification of bacterial *16S rRNA* and *groEL* genes by using the primers listed in Table 1^20^,^39^,^40^. PCR reactions were conducted under a temperature profile of 95 °C for 10 min, followed by 35 cycles of 95 °C for 30 sec, 55 °C (in the cases of *16S rRNA* and *groEL* bacterial gene) or 48 °C (for mitochondrial *COI* gene) for 1 min and 72 °C for 1 min, and a final extension at 72 °C for 10 min. The PCR products were subjected to agarose (1%) electrophoresis and fragments with the expected size excised from gel, purified and sequenced from both ends using four replications.

#### Molecular phylogenic analysis

To develop phylogenetic trees of the insect and the symbiotic bacterium, multiple sequence alignments were performed by using clustalX v.2 program^41^. Molecular phylogenetic analyses based on maximum likelihood and neighbor-joining algorithms were created by MEGA 6.06^42^. GTR + G + I model was selected as the best-fit substitution model for the maximum likelihood trees of *16S rRNA* and *COI* genes, and GTR model was used for *groEL* trees. The Bootstrap values were calculated with 1000 replications for neighbor-joining method and 100 replications for maximum likelihood method.

#### Co-evolutionary analysis

Tanglegram algorithm was used for co-phylogeny analyses between the host and the symbiont. We utilized Dendrescop software^43^ for constructing tanglegram by using maximum Likelihood trees of the host and the symbiont. I(cong) index was used for testing the topological congruence between trees^44^.

#### Light and electron microscopy

To observe bacterial symbiont cells inside the insect host crypts, the forth section of the insect midgut was fixed in 2.5% glutaraldehyde, 4% formaldehyde in 0.1 M sodium cacodylate buffer pH 7.2. Then, the material was rinsed three times in the same buffer, post-fixed in 2% osmium tetroxide and 0.8% potassium ferricyanide for 2 h. Following fixation, samples were dehydrated in a graded acetone series and embedded in epon–araldite resin^45^. Ultra-thin sagittal sections (60–70 nm) of the midgut were cut with an ultramicrotome and mounted on copper grids. The samples were stained in uranyl acetate and lead citrate and analyzed in a Philips CM30 300 kv transmission electron microscope (TEM). For light microscopy analyses the samples were cut at 5 μm thickness in a Minot microtome, stained with 0.5% toluidine blue and analyzed in a light microscope Olympus BH2.

#### Symbiont elimination

Surface sterilization of eggs was used for elimination of the bacterial symbionts. Six groups of the egg mass were treated with 70% ethanol for 10 min, followed by 10% hypochlorite sodium for different times (15 second, 30 second, 3 min, 5 min, 7 min, and 14 min). Finally, the treated egg masses were rinsed with 70% ethanol, whereas the control egg masses were treated with sterile water only. To confirm successful removal of the symbiont, DNA extraction was performed from the controls and the surface sterilized eggs. Then, DNA samples were used for PCR-based detection of bacterial symbiont.

#### Quantitative PCR (qPCR)

We used qPCR-based approach to analyze bacterial community structure at broad taxonomic levels. To do this, eight taxon-specific primer sets were used to quantify the abundances of the dominant groups of bacteria within the insect gut. The selected primer sets target the major phylogenetic groups of bacteria (Table 1)^46^,^47^. Moreover, the specific qPCR primers of the bacterial *groEL* gene were designed based on *groEL* sequence that was obtained in this study. Specific qPCR primers were also designed based on conserved regions of 18S rRNA gene in Pentatomidae insects and used as reference gene. The sequences of all primers were noted in Table 1.

To assess the bacterial community structure in along the insect gut, DNA was extracted from each region of the gut i.e. V1-V4. For quantification of the bacterial symbionts in different developmental stages of the insect, DNA was extracted from the first, second, third, fourth and fifth instar nymphs of *G. lineatum*. To compare density of bacterial symbiont in the aposymbiont and the control insects, DNA was extracted from surface-sterilized eggs, aposymbiont first instar nymphs, the control eggs and the control first instar insects. These samples were individually subjected to quantitative PCR (qPCR) using primers listed in Table 1. Genomic DNA was used for each qPCR using SYBR green (Ampliqon) with a micPCR instrument (BMS). PCR conditions were 95 °C for 15 min, followed by 40 cycles of 95 °C for 15 s, 15 s at the annealing temperature, and 72 °C for 20 s, followed by the melting curve (68–95 °C). Annealing temperatures are given in Table 1.

#### Analysis of the insect biology parameters

The offspring hatched from the eggs that were sterilized by hypochlorite sodium for 15 seconds were selected to assess additional biological parameters of the insect. The individuals hatchlings from the experimental egg masses along with the control insects were reared under laboratory condition at 27 ± 1 °C, 60 ± 5% RH and photoperiod of 16:8 h (Light: Dark). The insects were checked daily until final mortality was observed. Longevity, fecundity, pre-adult and adult stage duration rates of the insects were compared in the aposymbiotic and the control insects. These parameters in both groups of the insects were compared using Kruskal-Wallis and Mood-median tests and analyzed using SPSS. The graphs were created by using Graph Pad Prism.

#### Nucleotide sequence accession numbers

The nucleotide sequences of the bacterial *16S rRNA* gene, the bacterial *groEL* gene, and the mitochondrial *COI* gene of *G. lineatum* were deposited in the GenBank under accession numbers KR778999, and KR778997 and KR778998 respectively.

## Additional Information

**How to cite this article**: Karamipour, N. *et al*. Gammaproteobacteria as essential primary symbionts in the striped shield bug, *Graphosoma Lineatum* (Hemiptera: Pentatomidae). *Sci. Rep.* **6**, 33168; doi: 10.1038/srep33168 (2016).

## Figures and Tables

**Table 1:** Primers used in this study.

Target gene/Bacterial group	Primer	Primer sequence (5′ — 3′)	Fwd./Rev	Annealing temp. (°C)	Ref.
*16s rRNA*	16SA1	AGAGTTTGATCMTGGCTCAG	Fwd.	55	40
	16SB1	TACGGYTACCTTGTTACGACTT	Rev.		
*GroEL*	Gro-F1	ATGGCAGCWAAAGACGTAAATTYGG	Fwd.	55	22
	Gro-R1	TTACATCATKCCGCCCATGC	Rev.		
*COI*	LCO1490	GGTCAACAAATCATAAAGATATTGG	Fwd.	48	39
	HCO2198	TAAACTTCAGGGTGACCA AAAAATCA	Rev.		
Universal bacterial	906F	AAACTCAAAKGAATTGACGG	Fwd.	61.5	47
	1062R	CTCACRRCACGAGCTGAC	Rev.		
α-proteobacteria	α682F	CIAGTGTAGAGGTGAAATT	Fwd.	61.5	47
	908αR	CCCCGTCAATTCCTTTGAGTT	Rev.		
β-proteobacteria	Eub338	ACTCCTACGGGAGGCAGCAG	Fwd.	61.5	46
	Bet680	TCACTGCTACACGYG	Rev.		
γ-proteobacteria	1080γF	TCGTCAGCTCGTGTYGTGA	Fwd.	61.5	46
	γ1202R	CGTAAGGGCCATGATG	Rev.		
Actinobacteria	Act920F3	TACGGCCGCAAGGCTA	Fwd.	61.5	47
	Act1200R	TCRTCCCCACCTTCCTCCG	Rev.		
Bacteriodetes	798cfbF	CRAACAGGATTAGATACCCT	Fwd.	61.5	47
	Cfb967R	GGTAAGGTTCCTCGCGTAT	Rev.		
Firmicutes	Lgc353	GCAGTAGGGAATCTTCCG	Fwd.	61.5	47
	Eub518	ATTACCGCGGCTGCTGG	Rev.		
*18s rRNA*	penta-18srRNA-F	CCTGCGGCTTAATTTGACTC	Fwd.	55	This study
	penta-18srRNA-R	AACTAAGAACGGCCATGCAC	Rev.		
*GroEl*	Glsym-*groEL*-F	TTTCTAACGCCGGTGAAGAG	Fwd.	55	This study
	Glsym-*groEL*-R	ACCGAAGTCGATCATGTTGC	Rev.		
