# Corrigendum: Gammaproteobacteria as essential primary symbionts in the striped shield bug, *Graphosoma Lineatum* (Hemiptera: Pentatomidae)

**DOI:** 10.1038/srep40271

**Published:** 2017-01-23

**Authors:** Naeime Karamipour, Yaghoub Fathipour, Mohammad Mehrabadi

Scientific Reports 6: Article number: 3316810.1038/srep33168; published online: 09 09 2016; updated: 01 23 2017

The original version of this Article contained an error in the order of author names, which were incorrectly given as ‘Naeime Karamipour, Mohammad Mehrabadi & Yaghoub Fathipour’.

These errors have now been corrected in the HTML and PDF versions of this Article.

